# Analysis of the Emitted Wavelet of High-Resolution Bowtie GPR Antennas

**DOI:** 10.3390/s90604230

**Published:** 2009-06-03

**Authors:** Fernando I. Rial, Henrique Lorenzo, Manuel Pereira, Julia Armesto

**Affiliations:** Natural Resources & Environmental Engineering, EUET Forestal, University of Vigo, Campus A Xunqueira s/n. 36005 Pontevedra, Spain

**Keywords:** bow-tie antenna, source wavelet, time and frequency analysis, time zero

## Abstract

Most Ground Penetrating Radars (GPR) cover a wide frequency range by emitting very short time wavelets. In this work, we study in detail the wavelet emitted by two bowtie GPR antennas with nominal frequencies of 800 MHz and 1 GHz. Knowledge of this emitted wavelet allows us to extract as much information as possible from recorded signals, using advanced processing techniques and computer simulations. Following previously published methodology used by Rial *et al.* [1], which ensures system stability and reliability in data acquisition, a thorough analysis of the wavelet in both time and frequency domain is performed. Most of tests were carried out with air as propagation medium, allowing a proper analysis of the geometrical attenuation factor. Furthermore, we attempt to determine, for each antenna, a time zero in the records to allow us to correctly assign a position to the reflectors detected by the radar. Obtained results indicate that the time zero is not a constant value for the evaluated antennas, but instead depends on the characteristics of the material in contact with the antenna.

## 1. Introduction

**Figure 1.**
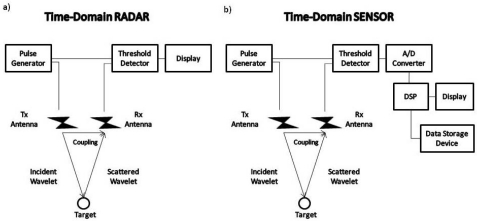
Classification of time domain radars according to the information obtained from the signal received. (a) Radar's schematic layout. (b) Sensor's schematic layout.

The term *radar* is short for radio detection and ranging. The name reflects the importance placed by the early workers in this field on the need for a device to detect the presence of a target and to measure its range. It should be noted that radar engineers use the term range to mean distance, a definition not found in some dictionaries [2]. Although the detection of range is still one of the most important functions of modern radars, these devices can extract much more information from a target's echo signal than its position. In this sense, some radars are often reclassified as sensors [3]. The main difference between a radar and a sensor lies in the receiver chain: radars usually have amplitude peak detectors whereas sensors preserve the entire signal for further analysis [4]. Analysis of the entire signal provides valuable additional information (Figure 1).

A common requirement in radars is a significant bandwidth, which allows for higher resolution capabilities of the equipment. For Ground Penetrating Radars (GPR), this bandwidth should be at least equal to the emission frequency of the antenna [5]. Because of this, most GPR devices employ wavelets or multi-frequency short-time pulses emitted in baseband without an intermediate carrying frequency. Such GPR systems are referred to as time domain systems.

Characterization of the wavelet emitted by the antennas is essential, as the pulse received by the radar is distorted and attenuated due to the propagation medium. Therefore, in order to make a good interpretation of the GPR data and extract as much information as possible from the signal recorded during processing, a deep knowledge of the type of emission used is important because the characteristics of the detected reflections (length and shape of the reflected pulse, overlapping of constructive or destructive reflections, etc.) and system's vertical resolution, directly depend on the characteristics of the wavelet emitted by the antennas [6,7]. In addition, advanced processing techniques such as deconvolution or specific algorithms for target recognition require specific knowledge of this signal for proper operation. Within the field of numerical simulation, it is also useful to work with the real source wavelet of the system. The goal of the simulation is to obtain a synthetic record very similar to that obtained in the field, which could aid in data interpretation. To provide practical results, modulation schemes in computer simulators should be able to incorporate, in addition to real antenna configurations and appropriate descriptions of the material properties, a precise model of the signal emitted by the antennas [8].

It is important to note that, despite the widespread commercialization of GPR, much of the antenna construction process is still done by hand. Therefore, antennas from the same company and with the same nominal frequency can differ slightly in terms of the emitted wavelet or radiation pattern.

As noted earlier, one of the important features of GPR equipment is the high resolution, especially within the first few meters, where medium-to-high frequency antennas are applicable. Some quality control experiments conducted on civil engineering structures or roads require centimetre, and sometimes millimetre, accuracy in both vertical and horizontal planes [9,10]. In GPR systems, a precise horizontal positioning is usually provided by an odometer, commonly a survey wheel. This device should be compatible with the system and software used for data acquisition and properly calibrated. The calibration should preferably be based on a long distance, in order to minimize errors in horizontal positioning. An accurate vertical positioning is determined by both an adequate setting of the time zero of the two-way travel time scale and a proper estimation of the medium velocity in which the signal propagates. This velocity will provide a correlation between obtained signal's propagation time and the range.

Previous studies have shown that the time zero of the radargrams is not necessarily a fixed value, as it depends not only on each particular antenna and the distance between transmitter and receiver, but also on the electromagnetic properties of the medium located just beneath the antenna [11,12].

To better understand our system and the emission characteristics of two bow-tie GPR antennas with nominal frequencies of 800 MHz and 1 GHz, we have conducted a series of tests following a methodology proposed by Rial *et al.* [1]. As a result, we performed a detailed analysis of the emitted wavelet in the time and frequency domains. In addition, according to the recommendations made by Yelf [10], a time zero is determined for each antenna in order to improve accuracy in range estimations of the reflectors detected by the radar.

## 2. Antenna Characteristics

**Figure 2.**
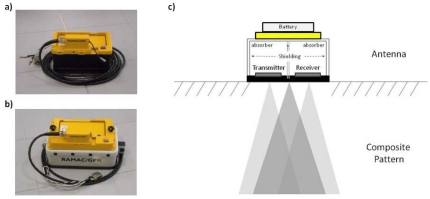
Antennas under test. (a) 1 GHz antenna. (b) 800 MHz antenna. (c) Composite radiation pattern of a ground-coupled shielded antenna.

The antennas under test (AUT) are two GPR shielded antennas with central frequencies of 800 MHz and 1 GHz respectively, manufactured by Mala Geoscience [Figures 2(a-b)]. These are ground-coupled biestatic antennas of the bowtie type.

Although each evaluated antenna appears to be a single unit, most GPR systems use separate antennas for transmission and reception, known as the biestatic configuration. This biestatic configuration is used because it is not yet possible to obtain ultra-fast transmit-receive switches that operate in the sub-nanosecond region with sufficiently low levels of isolation between transmit and receive ports [13]. The need to use separate transmit and receive antennas causes a convolution of the separate radiation patterns, forming a composite pattern [Figure 2(c)]. In this sense, the effective wavelet recorded is dependent on the characteristics of both dipoles, not only the transmitter. Because of the disposition of the dipoles inside the antenna, the direct signal between them will always be present in the radar trace.

**Figure 3.**
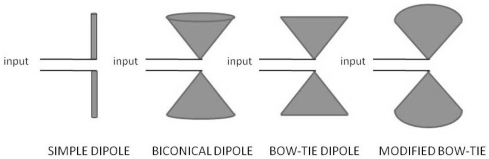
Geometric evolution from simple dipole antenna to bowtie.

Bowtie antennas are easy to manufacture and very popular within the GPR community. These antennas can be considered as an adaptation of a biconical antenna and ultimately evolved from a simple dipole (Figure 3).

Biconical antennas are excellent ultra-wideband radiators, but are usually not suitable for GPR because of their broad radiation pattern (low directivity) and design, thus making them impractical for fieldwork [14]. Bowtie antennas are a natural evolution of biconical antennas and are commonly employed in GPR due to their reasonably ultra-wideband properties and overall simplicity. The input impedance of bow-tie antennas is frequency independent for a given flare angle. This property is an attractive starting point for designing an adaptative antenna [15], but this dependence is usually eliminated by rounding the ends of the antenna [16]. There are other antenna designs that propose changes in the typical bowtie geometry in an attempt to improve different aspects of performance [17].

**Figure 4.**
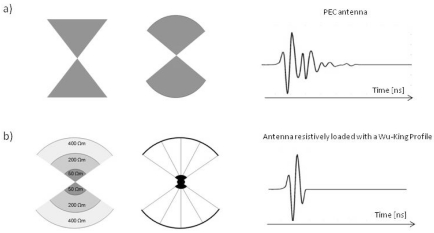
Examples of variation in the bowtie antenna design. (a) Bowties existing on almost perfect electric conductor (PEC) surfaces. (b) Bowties with resistive loaded profile to improve the late-time ringing. A design consisting of conductive wires of increasing resistance can be sometimes easier to implement.

Most bowtie antennas are designed and manufactured such that there is increasing resistance closer to the ends (Wu-King profile), which improves the resolution of the emitted wavelet (late-time ringing) (Figure 4). However, the benefits of this design are countered by a decreased in the efficiency of the antenna, with efficiency defined as the ratio of radiated power to input power. An alternative to resistive loading that does not decrease efficiency is capacity loading. This technique is not yet widely used and still requires investigation, but interesting designs that show promising performances have been proposed; either capacitive only, or in combination with microwave absorbers acting as resistors [18,19].

Bowtie antennas are usually formed by flat metal surfaces, but in attempt to surpass bowtie performance, designs based on longitudinal wires (from the source to the ends), radial strips and zig-zag shapes have been proposed by several authors [15,20] (Figure 4).

The particular characteristics of the bowtie antennas evaluated in this study are not routinely provided by the manufacturer and therefore remain unknown. However, the work of Valle *et al.* [21] provides information about the size and shape of the antenna dipoles within a 1 GHz antenna. The only parameter provided by the manufacturer is the internal distance between transmitter and receiver: 10 and 14 cm for the 1 GHz and 800 MHz antenna, respectively.

## 3. Methodology

**Figure 5.**
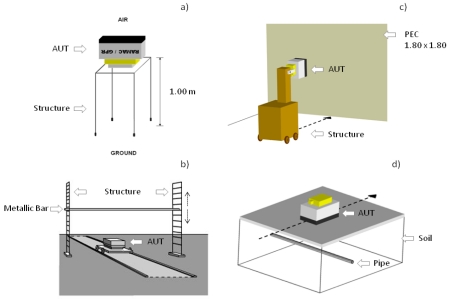
Experimental setups used to obtain: (a) the direct signal between dipoles in a reflector-free environment, (b) the emitted wavelet in air reflected in a metallic bar at different distances, (c) the emitted wavelet in air reflected in a PEC at different distances and (d) the emitted wavelet in a lossy dielectric reflected in a large buried pipe.

In order to conduct time and frequency analysis of the signal emitted by the antennas and accurately determine the time zero of the radargrams, we performed a series of tests following a simple methodology proposed by Rial *et al.* [1] (Figure 5). A basic knowledge of radar operation and access to some simple tools are sufficient in carrying out these tests. A spacious laboratory has proven to be adequate to test the high-frequency GPR antennas evaluated in this work.

As noted before, due to the use of two dipoles, one for transmission and the other for reception, the direct signal between both is always registered in any trace. A priori, this signal might be considered a possible approximation of the emitted pulse, but one must take into account both the proximity between the two dipoles and their arrangement inside the antenna (not opposite) [Figure 2(c)]; on the other hand, due to the antenna shielding, it is possible that some internal reflection can reach the receiver almost simultaneously in spite of the absorbent material, substantially varying the characteristics of the signal recorded in comparison with the emitted wavelet. Therefore, in order to study first the direct signal between dipoles, each antenna under test (AUT) was placed in an environment free of any reflectors that might overlap the registration of the signal. The direction of maximum radiation of the dipoles pointed into the air, leaving a plane containing both dipoles parallel to the ground [Figure 5(a)].

A methodology often used to achieve a very approximate version of the emitted signal is to obtain a reflection of this wavelet from an object or surface. Given its characteristics of linearity, homogeneity, isotropy, and non-dispersion, air becomes an ideal propagation medium in this case, ensuring that the waveform received is not affected by any of these effects that, to a greater or lesser extent, are present in all environments in which a study with a GPR is carried out. The main correction factor associated with this reflection is the geometric attenuation of the signal. This factor is also analyzed from the obtained results. According to this, two simple tests were performed with each AUT. The first is shown in Figure 5(c), where each antenna was placed against a wall that was covered with a perfect electric conductor (PEC). The dimensions of the PEC were 180 × 180 cm. The scheme followed in the second test is shown in Figure 5(b). In this case, we used a different reflector consisting of a metallic bar 3 cm in diameter. The metallic bar is placed in the plane of separation between the transmitter and receiver. In both tests (PEC and metallic bar), series of successive measurements were carried out, gradually varying the separation between the antenna and reflector. By taking multiple measurements instead of a single measurement, our results could be used to characterize both the geometric attenuation of the signal and the minimum distance of overlap between the direct signal and a first reflector.

To complement the tests in air, a third test was conducted over a paved area where a large pipe was known to be present [Figure 5(d)]. The goal of this experiment was to carry out a profile transverse to the longitudinal axis of the pipe in order to obtain a clear reflection of the emitted wavelet on the pipe. This experiment will help to elucidate the actual behaviour of antennas when working with common surveying materials and compare the obtained wavelets with those obtained in air. The complete description of these experimental devices and the laboratory procedure can be read in [1].

The data obtained following the methodology of Figure 5(c) was also used to calculate the time zero of the antennas. Since the distance between the antenna and the reflector is known, these tests allow us to establish a time zero in air. To complete the time zero analysis, a second group of tests were carried out following the same methodology but using different reflectors instead of a PEC (brick, concrete, wood, etc.). These experiments allow us to analyze the possible misalignment of the time zero arising from the influence of the medium.

Before starting each test, the AUT was subjected to a warm-up time of 10 min. During this warm-up time, the equipment was constantly acquiring data, triggered by time at fast speed. Previous tests with this equipment [22], showed that 10 min are enough to reach the maximum stability achievable with this particular system.

**Figure 6.**
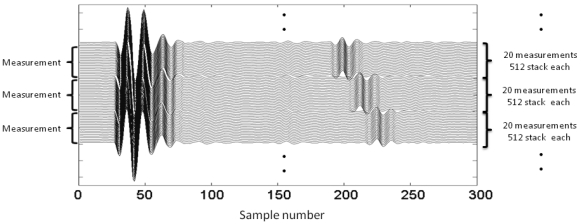
Example of measurements performed at different distances. For each distance, 20 measurements with 512 stacking each were obtained. Amplitude variations between measurements are close to 1%, whereas arrival time variations are practically negligible.

It is important to point out that most of GPR equipments use stacking to improve signal's stability and reduce noise power levels [23]. In this case, each trace gathered in the tests results from an average (stack) of 512 consecutive traces emitted and received by the system. In doing so, we guarantee sufficient stability in time and amplitude of the signal, according to the recommendations of Rial [22], Scullion *et al.* [24] or Manacorda *et al.*[23]. For every measurement, 20 traces with high stacking (512) were collected (Figure 6). Obtained amplitude variations are close to 1%, whereas arrival time variations are practically negligible.

## 4. Results

### 4.1 Emitted Wavelet and Direct Signal

**Figure 7.**
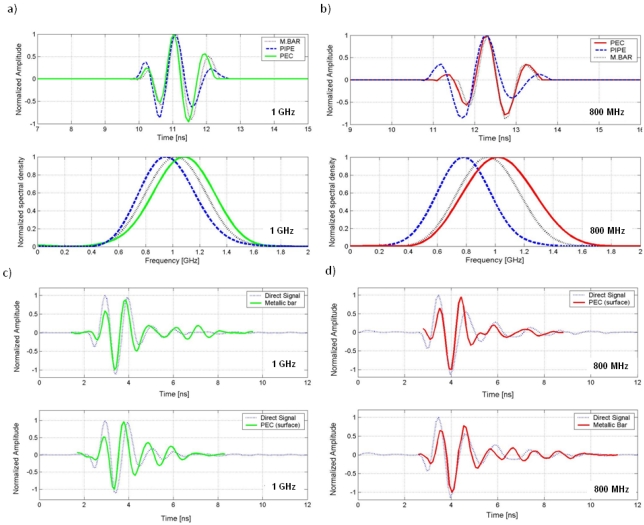
(a-b) Time and spectral domain comparison between wavelets obtained using the different methodologies for the 1 GHz and 800 MHz antennas, respectively. (c-d) Similarity between the direct signal and the emitted wavelet for the 1000 and 800 MHz antennas, respectively. (Emitted wavelets were normalized and inverted in phase for comparison with the direct signal).

Figures 7 (a-b) show the emitted wavelets obtained in the tests with the PEC surface, metallic bar and pipe with the 1 GHz and 800 MHz antennas, in time and frequency domain. Figures 7 (c-d) compare the direct signal between transmitter and receiver for each AUT for selected wavelets.

Table 1 indicates the time intervals between values of 1/2 (corresponding to 3 dB) and 1/10 (corresponding to 10 dB) with respect to the recorded maximum amplitude for each obtained wavelet and AUT. Table 2 and 3 contain similar parameters related to the frequency characterization of these wavelets.

**Figure 8.**
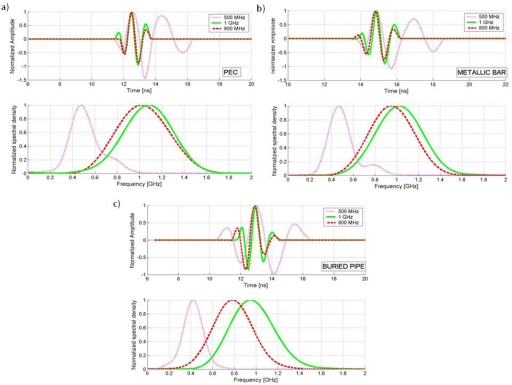
Comparison between wavelets obtained in the different tests. (a) PEC. (b) Metallic bar. (c) Buried Pipe. (Emitted wavelets of a 500 MHz antenna obtained in a previous work are also show for comparison).

Figure 8 shows a qualitative comparison between the wavelets obtained for each methodology. Additional wavelets from a 500 MHz antenna, obtained in a previous work using the same methodologies presented here [1], are shown for comparison.

**Figure 9.**
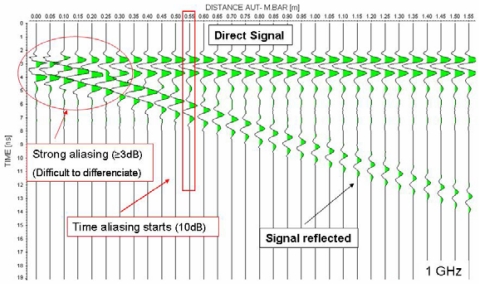
An obtained radargram where it is possible to observe the recorded wavelet after its reflection in the metallic bar at different distances for the 1 GHz antenna.

As discussed previously, for the reflection tests on the metallic bar and aluminium surface, a series of successive measurements were performed, gradually changing the separation between the AUT and the reflector. By conducting a series of measurements instead of a single measurement, we were able to study the distance of the minimum overlap between the direct signal and the reflection from a surface. Figure 9 shows the sequence of consecutive traces in the results obtained for different separations between the metallic bar and the 1 GHz antenna.

Table 4 provides the antenna-surface distances where the overlap between the reflection on the surface and the direct signal is at 3 and 10 dB levels. At distances greater than 10dB, a differentiated reflection of the surface can be obtained.

**Figure 10.**
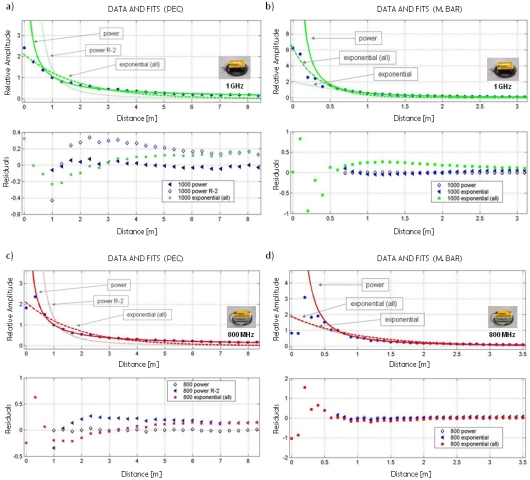
Maximum amplitude peaks of the reflected wavelet at different distances. For each methodology, three possible regression curves and their residual variations are shown. (a-b) PEC and metallic bar for the 1 GHz antenna. (c-d) PEC and metallic bar for the 800 MHz antenna.

From the results obtained using this methodology, it is also possible to study the attenuation of the reflected wavelet as a function of reflector and antenna separation. We use the peaks of maximum amplitude of the reflected wavelet for each position as the amplitude values in the study. Figure 10 shows the results obtained from the two AUT and corresponding regression curves. Tables 5 and 6 show the equations of these regression curves and the values of the variables that give the best fit for the obtained AUT data. We have also included fitting parameters related to the quality of the fit.

### 4.2. Time Zero Analysis

**Figure 11.**
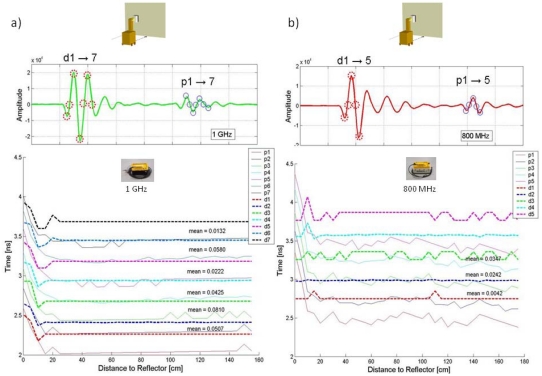
Time zero analysis from data obtained using the methodology shown in Figure 5(c). The figure displays the points taken as reference in each trace collected at different distances between the antenna and PEC. Each point represents a line shown in the lower part of the figure. The position of the reflected wavelet points are corrected according to the antenna-PEC distance at the time of the measurement. The average distance between closest lines is provided.

Figure 11 shows the results of the time zero determination using tests performed with the methodology shown in Figure 5(c).

A first approximation of the time zero was set using the reflection on the PEC surface at different distances. We established a time zero reference using time measurements between significant points in the direct signal and reflected wavelet. We chose our points with the knowledge that data processing programs are typically able to automatically locate maximum, minimum and zero cross points in the traces and perform accurate measurements between them.

The lower portion of Figure 11 shows the significant points of the reflected signal as dotted lines. The positions of these points are constant in the figure, as the direct signal does not change until there is a strong overlap between direct and reflected signals. In those positions, it is difficult to differentiate both signals. The significant points of the reflected signal are drawn as continuous lines. Since the distance between the reflector and the antenna is known, a correction factor is applied to each point, according to its distance. This graph, therefore, qualitatively shows the relationship between significant points in the direct and reflected signals. The average distance between closest lines is displayed. Interestingly, there are small, random variations in the positions of the significant points of the reflected wavelet after correction. Most likely, this is due to the small instability in amplitude (1%) of the system's signal that was commented before.

**Figure 12.**
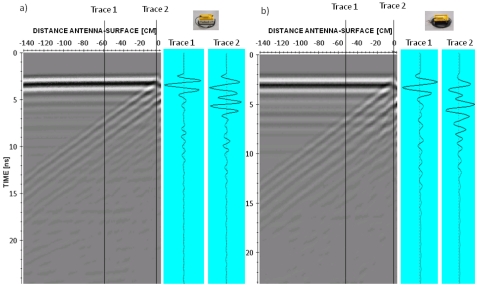
Example of time zero tests carried out following the methodology of Figure 5(c) over a typical brick wall of thickness 12 cm and wave velocity 11 cm/ns: (a) 800 MHz. (b) 1 GHz

Additional tests were made following the same methodology, but conducted over different materials with a known composition and thickness. These tests allow us to analyze the stability of the time zero criteria adopted for each AUT when antennas are close to the medium's surface. Figure 12 shows an example of the tests performed.

As in the experiments on the PEC, when the AUT is close to a surface, the direct signal is altered due to temporal aliasing with the reflection on the surface and associated multiples. This alteration is variable, depending on the properties of the medium and antenna distance. Changes in the direct signal amplitude are significant at certain distances from the surface where the combination with the reflected wavelet is more destructive or constructive.

As it can be appreciated comparing trace 1 and trace 2 in Figure 12, there is usually also a slight shift of the entire trace towards higher values in the time scale when the antenna is in contact to -or very near- a surface. This phenomenon is known as radiation delay and it has been documented by other authors using similar systems [12,25]. In addition, for the two evaluated antennas, it is frequently observed a slight increase of the DC component as the antenna gets closer to the surface of a medium.

## 5. Discussion

In this study, we were able to obtain an approximation of the wavelet emitted by two GPR ground-coupled shielded antennas of the bowtie type. The analysis in time of the length and shape of the wavelets has allowed for approximate characterization of these signals. The frequency domain analysis has revealed the frequency ranges used by the antennas and their spectral power distribution. Of significance is the similarity found between the emitted wavelets of both antennas when tests were performed on either the PEC or metallic bar. As illustrated in the comparative graphs of Figure 8, the appearance of both signals is nearly identical. On close inspection, the larger amplitude peaks of the 1 GHz wavelet lead to this wavelet having even wider time intervals in air than the 800 MHz one at 3 and 10 dB levels (Table 1). The central frequency of the 800 MHz wavelet in air is surprisingly high and actually closer to 1,000 MHz (Table 2).

Experiments carried out over the buried pipe help to elucidate the actual behaviour of antennas when working with common surveying materials. For both antennas, we observed a decrease in the central frequency and bandwidth: in the time domain, this is seen as a widening of the emitted pulse (Tables 1-3). This effect arises because of two reasons. First, the medium acts as a low-pass filter so that the pulse broadens in time, consequently narrowing its associated frequency band. Second, the proximity of the medium causes the frequency spectrum of the antennas to shift to lower frequencies.

We have determined the minimum distances between AUT and surface that still allow for a clear reflection of the latter without interfering with the direct signal (Table 4). In this sense, two approximate separation levels have been established. In the first level (10 dB), the separation is almost complete and overlap occurs only between the components of the pulse of lower energy. From the second level (3 dB), the higher energy components begin to overlap and it becomes very difficult to distinguish between the two signals.

To calculate the attenuation curves obtained from the amplitude peaks of the reflected signals in the PEC and metallic bar, we used the common term for characterization of the geometric expansion of a wavefront, 1/range^n^. For the tests with PEC, a value of n = 2 was used, equal to the theoretical value for a spherical wave and a plane reflector of this type [1]. It should be noted that this approach assumes that the object is considerably far from the antenna, which explains why the adjustment using this equation is not entirely accurate. All the values obtained for the n parameter are between 0.8 and 1.5, being remarkably similar in the two experiments with the 800 MHz antenna.

Although perhaps not initially intuitive, the time zero is not a constant value for the ground-coupled antennas, but instead depends on the characteristics of the material near the antenna. This situation, studied extensively by Yelf [12], is verified experimentally in the work presented here. In many GPR systems, the software automatically sets the zero at the beginning of the trace or after a certain number of samples, making necessary for the user to further adjust the position to obtain a precise travel time scale. Through a series of simple tests, we have shown it is possible to establish criteria for determining, in a practical way and with the least possible error, the time zero of evaluated antennas by using significant points in the direct signal and reflected wavelet.

In any case, GPR ground-coupled antennas are designed to be in contact or close to a medium's surface. Due to the influence of the medium on the direct signal when the antenna is near, it seems necessary to supplement the results with additional tests on the particular material under study, in order to determine a more accurate time zero. Setting a more precise zero time for a specific material can be difficult and sometimes unnecessary. It must be taken into account that, in practice, it is often difficult to determine the polarity of the reflected wavelet or identify which semi-period corresponds to a certain peak amplitude because the wavelets are modified by other reflections being recorded at the same time. The additional signal can, in some cases, make a wavelet's lower peaks have higher amplitudes than central ones. This difficulty can be circumvented through a careful analysis of the wavelet. Continuous reflectors, such as asphalt layers, are less prone to this type of error criterion. Another possibility to improve the accuracy of the zero adjustment is through deconvolution of the data. Using this analysis technique on tests performed over pavement has proven to provide interesting improvements in the accuracy of the calculation of layer thickness [26].

## Figures and Tables

**Table 1.** Time intervals between values of 1/2 (corresponding to 3 dB) and 1/10 (corresponding to 10 dB) with respect to the recorded maximum amplitude of the wavelet obtained in the different experiments for both antennas. Measurements were obtained with high stacking (512) to ensure adequate signal stability.

	**800 MHz**		**1 GHz**	

	**Δt_3dB_ (ns)**	**Δt_10dB_ (ns)**	**Δt_3dB_ (ns)**	**Δt_10dB_ (ns)**
**METALLIC BAR**	1.43	2.38	1.46	2.28
**PEC**	1.3	2.2	1.4	2.8
**BURIED PIPE**	1.44	2.8	1.49	2.51

**Table 2.** Frequency characteristics of the effective wavelets obtained for the 800 MHz antenna. Measurements were obtained with high stacking (512) to ensure adequate signal stability.

**800 MHz**	**f_central_ (air)** **(MHz)**	**3dB f_L_** **(MHz)**	**3dB f_H_** **(MHz)**	**BW_3dB_** **(MHz)**	**10dB f_L_** **(MHz)**	**10dB f_H_** **(MHz)**	**BW_10dB_** **(MHz)**
**BURIED PIPE**	785	568	998	430	398	1180	782
**METALLIC BAR**	950	704	1200	496	525	1390	865
**PEC**	1000	740	1310	570	550	1540	990

**Table 3.** Frequency characteristics of the effective wavelets obtained with the 1 GHz antenna. Measurements were obtained with high stacking (512) to ensure adequate signal stability.

**1 GHz**	**f_central_ (air)** **(MHz)**	**3dB f_L_** **(MHz)**	**3dB f_H_** **(MHz)**	**BW_3dB_** **(MHz)**	**10dB f_L_** **(MHz)**	**10dB f_H_** **(MHz)**	**BW_10dB_** **(MHz)**
**BURIED PIPE**	960	717	1200	483	552	1410	852
**METALLIC BAR**	1000	740	1310	570	550	1540	990
**PEC**	1008	818	1340	522	595	1540	945

**Table 4.** Minimum distance between antenna and surface to obtain a reflected wavelet at different overlapping degrees. Measurements were obtained with high stacking (512) to ensure adequate signal stability.

	**Direct Signal – Surface 3dB aliasing**	**Direct Signal – Surface 10dB aliasing**
**1 GHz**	30 cm (1.16λ)	60 cm (2λ)
**800 MHz**	30 cm (1.1λ)	75 cm (2.37λ)

**Table 5.** Values that allow the best fit of the regression curves for the 800 MHz antenna. Measurements were obtained with high stacking (512) to ensure adequate signal stability. Concerning the quality of the fit, sum of squared errors (SSE) and correlation coefficient (R-square) parameters are included in the table.

	**Type**	**Equation**	**Range**	**a**	**b**	**SSE**	**R-square**

**800 MHz** **(M.bar)**	Power	*a x^b^*	x ≥ 60cm	1.384	-1.297	0.01	0.99
	Exponential	*a e^bx^*	x ≥ 60cm	2.28	-0.6677	0.08	0.93
	Exponential (all)	*a e^bx^*	all	6.746	-1.368	5.22	0.64

**800 MHz** **(PEC)**	Power	*a x^b^*	x ≥ 60cm	1.004	-0.8802	0.004	0.996
	Power (2)	*a x^-2^*	x ≥ 60cm	1.338	-2	0.86	0.18
	Exponential (all)	*a e^bx^*	all	2.07	-0.5471	0.81	0.89

**Table 6.** Values that allow the best fit of the regression curves for the 1 GHz antenna. Measurements were obtained with high stacking (512) to ensure adequate signal stability. Concerning the quality of the fit, sum of squared errors (SSE) and correlation coefficient (R-square) parameters are included in the table.

	**Type**	**Equation**	**Range**	**a**	**b**	**SSE**	**R-square**

**1 GHz** **(M.bar)**	Power	*a x^b^*	x ≥ 60cm	0.5889	-1.509	0.0005	0.9996
	Exponential	*a e^bx^*	x ≥ 60cm	2.05	-1.174	0.03	0.97
	Exponential (all)	*a e^bx^*	all	6.207	-2.874	2.83	0.95

**1 GHz** **(PEC)**	Power	*a x^b^*	x ≥ 60cm	1.068	-0.8336	0.02	0.97
	Power (2)	*a x^-2^*	x ≥ 60cm	1.441	-2	1.23	0.002
	Exponential (all)	*a e^bx^*	all	2.089	-0.5231	0.45	0.93
